# The carbonization of polyacrylonitrile-derived electrospun carbon nanofibers studied by *in situ* transmission electron microscopy†

**DOI:** 10.1039/c8ra10491c

**Published:** 2019-02-21

**Authors:** Roland Schierholz, Daniel Kröger, Henning Weinrich, Markus Gehring, Hermann Tempel, Hans Kungl, Joachim Mayer, Rüdiger-A. Eichel

**Affiliations:** Forschungszentrum Jülich GmbH, Institute of Energy and Climate Research, Fundamental Electrochemistry (IEK-9) Germany r.schierholz@fz-juelich.de; Institute of Physical Chemistry, Rheinisch Westfälische Technische Hochschule Aachen Germany; Jülich Aachen Research Alliance: JARA-Energy Germany; Forschungszentrum Jülich GmbH, Ernst Ruska-Centre (ER-C) for Microscopy and Spectroscopy with Electrons Germany; Central Facility for Electron Microscopy, Rheinisch Westfälische Technische Hochschule Aachen Germany

## Abstract

Cathode structures derived from carbonized electrospun polyacrylonitrile (PAN) nanofibers are a current line of development for improvement of gas diffusion electrodes for metal–air batteries and fuel cells. Diameter, surface morphology, carbon structure and chemical composition of the carbon based fibers play a crucial role for the functionality of the resulting cathodes, especially with respect to oxygen adsorption properties, electrolyte wetting and electronic conductivity. These functionalities of the carbon fibers are strongly influenced by the carbonization process. Hitherto, fibers were mostly characterized by *ex situ* methods, which require great effort for statistical analysis in the case of microscopy. Here, we show the morphological and structural evolution of nanofibers during their carbonization at up to 1000 °C by *in situ* transmission electron microscopy (TEM). Changes in fiber diameter and surface morphology of individual nanofibers were observed at 250 °C, 600 °C, 800 °C and 1000 °C in imaging mode. The structural evolution was studied by concomitant high resolution TEM and electron diffraction. The results show with comparatively little effort shrinkage of the nanofiber diameter, roughening of the surface morphology and formation of turbostratic carbon with increasing carbonization temperature at identical locations.

## Abstract 2

The carbonization of polyacrylonitrile (PAN) nanofibers is observed *in situ* up to 1000 °C by transmission electron microscopy (TEM).

## Graphical abstract

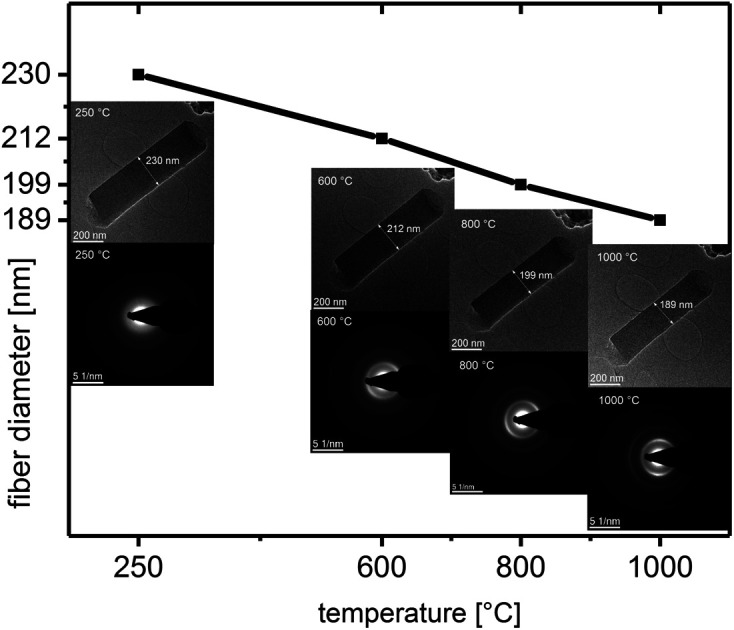

## Introduction

Electrospinning is an efficient technique to provide 1-D nanostructured polymeric or polymer-based materials and composites.^1^ Recent research identified carbonized polymer-derived fibers as a promising class of materials for a wide scope of energy applications, such as in catalyst supports for direct methanol fuel cells, methanol oxidation and water splitting.^2–4^ The catalytic activity for the oxygen reduction reaction in alkaline media makes nitrogen doped carbon materials a promising candidate for cathode structures of metal air batteries.^5^ In addition polyacrylonitrile (PAN)-derived carbon fibers also find application in supercapacitors and as anode structures in lithium-ion batteries.^6–8^ The tunability of the fiber properties relevant for their functionality – wettability, porosity of fiber surfaces, chemical composition and structure of the carbonized fibers – underline the potential of this type of material.^9^ However, a precondition for the tailoring of properties is knowledge and control over the effects of process specifications during the preparation of the polymer solutions, the electrospinning process as well as the crosslinking and the carbonization steps.

After electrospinning the standard procedure to convert polymeric PAN-fibers into carbon fibers is oxidative stabilization, carbonization and graphitization.^10^ The first of these three steps is performed under air between 200 °C and 300 °C and the reactions involved are the cyclisation of nitrile and incorporation of oxygen as described by Goodhew *et al.*^11^ This step is important to avoid votalilization, maximize the carbon yield and avoid the formation of hollow core fibers in the subsequent carbonization step.^10,12^ The subsequent carbonization step is conducted under inert gas atmosphere up to temperatures of about 1500 °C. For higher temperatures, generally above 2500 °C, the term graphitization is used.^10^ Graphitization is usually performed for “high modulus” carbon fibers, used for reinforced plastics and not discussed in this manuscript, which focuses on carbonization below 1500 °C. The carbonization step involves losses of oxygen and nitrogen, still present after crosslinking. The corresponding evaporation processes start at temperatures just above the stabilization temperature of *e.g.* 400 °C.^13^ However, most changes were reported for temperatures between 700 °C and 1200 °C.^12^ Consequently changes in chemistry and structure of the fibers, but also in their dimensions and surface morphology result from the carbonization step in this temperature range.

A wide scope of analytical techniques such as X-ray Photoelectron Spectroscopy (XPS), X-Ray Diffractometry (XRD), Raman, infrared-spectroscopy, thermogravimetry (TGA) and differential thermoanalysis (DTA) has been applied for the characterization of structure, crosslinking/stabilization and carbonization behavior of PAN-derived carbon fibers.^7,14–20^ While most of these methods provide an overall view on the fiber materials, a technique of choice in order to obtain localized information – in particular on the fiber surfaces – is transmission electron microscopy (TEM).

Most TEM-studies were performed to study strength structure-relationship of carbon microfibers. In 1976 for example Bennett^21^ did an extensive three-dimensional analysis of PAN-derived carbon fibers heat treated at 1000 °C, 1500 °C and 2500 °C using various TEM techniques such as bright and dark field TEM, high resolution transmission electron microscopy (HRTEM) and quantitative electron diffraction analysis on transverse and longitudinal cross-sections. A more recent HRTEM investigation of copolymerized acrylonitrile/itaconic acid fibers drawn from a spinneret in a coagulation bath is presented by Bai *et al.*^22^ Here, amorphous and ordered structures were identified even without heat treatment. Furthermore, while highly oriented structures were detected in the longitudinal sections, the cross-sections showed onion like spherical ordering as well as crystallites.

Laffont *et al.* performed HRTEM, electron energy loss spectroscopy (EELS) and XRD on different types of commercial PAN-derived carbonized microfibers. They report coherent turbostratic graphite with a *d*-spacing larger than 3.43 Å.^23^ This is larger than the value of 3.35 Å published for graphite.^24,25^ Moreover, the size of the stacks firstly remains small with *L*_10_ ≤ 4 nm (parallel to the graphitic planes) and *L*_002_ ≤ 1.3 nm (perpendicular to the graphitic planes), but increases with processing temperature from 300 °C to 1000 °C.^23^ EELS analysis of the K-edges of carbon, nitrogen and oxygen shows that a carbon content of 99% is achieved for carbonization at 1000 °C, while only little amount of oxygen (0.5–1% from initially 1–4%) remains in the fibers.^23^ More dramatic is *I* the change of nitrogen content from initially 1–12% it drops to zero after 1000 °C, after 800 °C 1–3% are reported.^23^ In addition Laffont *et al.* also studied the σ + π plasmon in the low loss region and show that the σ-plasmon shifts to higher energies for materials processed at higher temperature and correlate this with lower resistivity.^23^ In a later work Laffont *et al.* used a combination of EELS and XPS to study the bonding situation especially of nitrogen.^26^ In this study, they report an increase of about +7 at% during stabilization in air at 250 °C and a loss of up to −15 at% during subsequent carbonization at up to 1000 °C in N_2_-atmosphere. Depending on the fiber treatment after spinning significant amounts of nitrogen [N]/[C] ≈ 0.1 and oxygen [O]/[C] ≈ 0.05 remain in the fiber. The chemical composition and bonding situation of nitrogen in carbon fibers were also studied in detail by other groups using XPS to distinguish different bonding types of nitrogen for application in batteries and fuel cells.^2,14,19^

The previously described results were mostly related to microfibers with diameters ranging from 5 to 12 μm. Musiol *et al.* compared PAN-derived nano and microfibers heat treated at 1000 °C, 2000 °C and 2800 °C, and reported higher massloss for nanofibers, 61% residual mass for carbon microfibers and 45% residual mass for carbon nanofibers after a heat treatment at 1000 °C.^18^ HRTEM and Raman spectroscopy showed graphitization for fibers treated above 2000 °C while the 1000 °C fibers still appear quite amorphous.^18^ For the temperature range from 1500 °C to 2800 °C a development from relatively smooth to rough and ridged morphology was reported by Kurban *et al.*^27^ In all reports temperature ranges differ and also a variety of different investigation techniques is applied. It is shown that the change of chemical composition; carbon structure and morphology of the fibers strongly depend on the carbonization process as well as the treatment applied in the previous steps.^18,26,27^

Common feature of most microscopy investigations is that the comparison of structural and morphological characteristics was performed based on *ex situ* experiments. Indeed, *ex situ* analyses are advantageous with respect to flexibility of the fibers processing conditions as they are not limited by the *in situ* experiment. However, comparisons of fibers subject to small process variations require that the effects of the process variation exceed the scattering in characteristics of individual fibers. Otherwise, large efforts by statistical analysis of many samples are required to provide significant data for establishing correlations between processing parameters and structural as well as microstructural characteristics. An approach to circumvent this is, to investigate the evolution of fiber characteristics on identical locations on individual fibers during processing with *in situ* microscopy methods. A first attempt was applied by Prilutsky *et al.* for the case of the carbonization of electrospun PAN nanofibers containing carbon nanotubes in 2010.^28^ However, in their study a heating stage, where the whole 3 mm grid is heated up, was used. Due to their larger heating volume such heating stages as a result feature slow response times, inaccurate temperatures, sample instabilities and strong thermal drift, thus limit the TEM image resolution. In this study we aim on studying the shrinkage of fiber diameter, surface morphology as well as structural changes on the same fibers in one single *in situ* TEM heating experiment at four subsequent temperature stages – 250 °C, after oxidative stabilization, 600 °C, 800 °C and 1000 °C on an *in situ* heating holder based on micromechanical systems (MEMS).^29^ In contrast to the before mentioned system this *in situ* holder allows high resolution TEM images and electron diffraction in accurate temperatures without instabilities, which will help to acquire a conclusive picture for the evolution of electrospun PAN nanofibers during carbonization.

## Experimental

### Materials preparation

PAN nanofibers were prepared from a dimethylformamide (DMF) solution >99% (Sigma Aldrich) containing 10 wt% PAN (molecular weight 150 000, Sigma Aldrich) using an IME EC-CLI (IME, Netherlands) electrospinner. The device was setup with a 0.8 mm nozzle diameter, a rotating cylindrical target (diameter = 20 mm) and a nozzle-to-target distance of 160 mm. An electric field of 15 kV was applied between nozzle and collector in a processing chamber, which was kept at 25 °C and 20% relative humidity. The nozzle and the target were operated at lateral movement of 20 mm s^−1^ within a range of 100 mm and a rotation speed of 1500 rpm respectively. The feed rate for the polymer solution was 0.02 ml min^−1^. The process was kept running for 30 min resulting in polymeric nanofiber mats of 100 mg with dimensions of 60 × 100 × 0.05 mm approximately. The polymeric nanofiber mats were dried in a cabinet at 200 °C overnight to evaporate the remaining solvent. Oxidative stabilization and partial cross-linking was performed in air at 250 °C for 4 h.

### SEM characterization

Fiber mats were characterized by SEM (FEI, Quanta FEG 650) using an acceleration voltage of 2 kV. A micrograph of an oxidatively stabilized, non-carbonized fiber mat is shown in Fig. S1.† Fiber diameters were measured manually with Olympus Stream Essentials Desktop 1.9.3.

### TEM sample preparation

A dispersion of nanofibers in their oxidative stabilized state, after the crosslinking step at 250 °C, was prepared by ultrasonic treatment of a piece of the nanofiber mat in pure ethanol. A droplet of the dispersion was applied to the heating chip of a MEMS based *in situ* heating holder (DENSsolutions) with Si_3_N_4_ membrane and carbon coated windows. By means of focused ion beam holes of circular shape were previously etched into the carbon film to provide areas without carbon support for the experiment.^30^

### *In situ* carbonization conditions

**Fig. 1:**
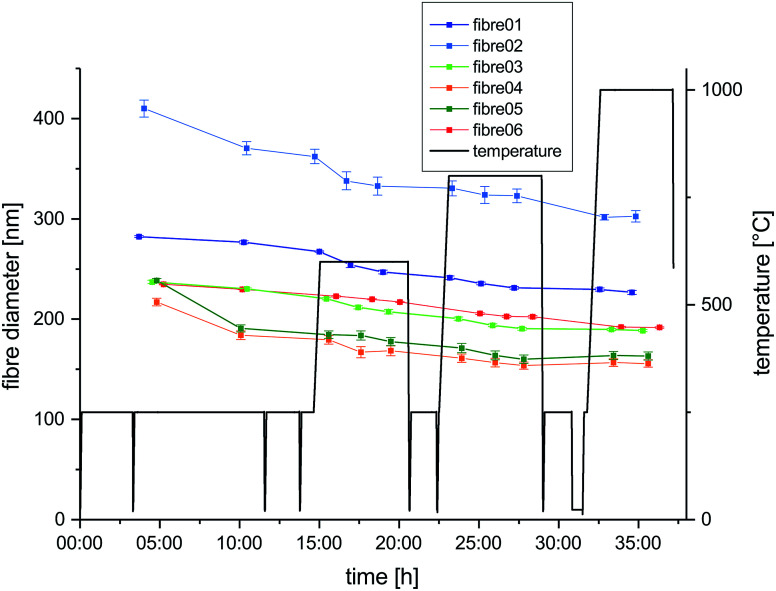
Heating program applied during experiment (black) and the measured diameters at different stages of the experiments for six selected nanofibers are plotted as a function of time (colored).

The temperature – time profile applied for the carbonization process during the *in situ* experiment is depicted in Fig. 1. The profile is designed for nanofiber characterization during four subsequent temperature stages – 250 °C, 600 °C, 800 °C and 1000 °C. All fibers located on the heating chip were heated simultaneously. Heating rate applied during all temperature steps was 15 °C min^−1^. Dwell times at the individual temperature steps were varying between 5 and 7 h according to the time required for TEM image acquisition of all nanofibers. In total, the *in situ* experiment was carried out on six fibers with three observations during each of the four different temperature steps. The temperature steps were split into several days of microscope time.

For three fibers (1, 3 and 6) investigations were limited to low resolution size analysis, which is supposed to involve only minor influence of the electron beam. Three other fibers (2, 4 and 5) were subject to high resolution imaging and diffraction, which implies substantially higher dose for the high resolution and longer exposure to the beam during the alignment of the microscope for electron diffraction. The atmosphere during the *in situ* carbonization was imposed by the ultra-high vacuum (UHV) conditions required in the TEM.^31^ After every heating period the sample holder was cooled down to ambient temperature by switching off the heating.

### TEM experiments

*In situ* TEM experiments were carried out on a FEI Titan with a spherical aberration (*C*_s_) corrector (CEOS) for the objective lens and operated at 300 kV using the negative-*C*_s_ imaging technique, which provides images with high contrast and low noise.^31,32^ In imaging mode the evolution of fiber diameters, fiber morphologies and alignment of the graphite layers was analyzed by low, intermediate and high resolution images respectively. The high resolution images were taken from lateral and fracture surfaces of the nanofibers. Diffraction patterns were taken from one nanofiber at a camera length of *L* = 490 mm where the {002} carbon reflection is not covered by the beam stop and the second and third diffraction rings are still visible. Images with the same settings were taken two to three times every two hours during each temperature step.

## Results

### Fiber diameter

**Fig. 2:**
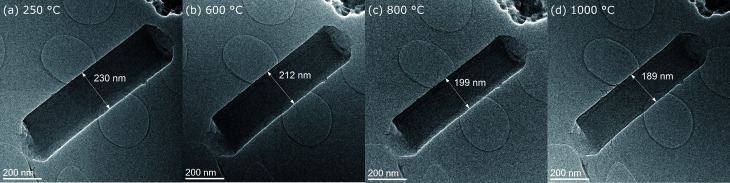
Overview images of fiber 6 at different stages during the *in situ* experiment (a) 250 °C, (b) 600 °C, (c) 800 °C and (d) 1000 °C.

Fiber diameters were measured on six different nanofibers at different stages in course of the *in situ* experiment and the evolution of their diameters over time is plotted together with the temperature profile in Fig. 1. All selected nanofibers have initial diameters in a range from 200 nm to just above 400 nm, which is typical for the processing used and in agreement with the fiber diameter distribution measured from SEM micrographs of the fiber mats (Fig. S1 and S2†). The evolution of the diameter of fiber 6 along with the subsequent temperature treatments at 250 °C, 600 °C, 800 °C and 1000 °C is shown exemplary in low magnification TEM images in Fig. 2(a–d). The shrinkage behavior is representative for a nanofiber carbonized under the influence of temperature and UHV, but largely unaffected by the electron beam. Very similar changes in diameters are observed for fiber 1 and 3, with minimized exposure to the electron beam. The decrease in diameter over the complete cycle is in the range between 15 and 20% compared to the initial diameter. In contrast to that, the three nanofibers exposed to the influence of the electron beam for extended time spans (fibers 2, 4 and 5) show larger shrinkage in diameter mounting up to 32%. This behavior is independent of the initial nanofiber diameters (Fig. 1). The enhanced shrinkage under the influence of the electron beam occurred in particular during the 250 °C annealing step in the microscope. At higher temperature, no significant differences between the shrinkage rates can be identified.

### Nanofiber morphology

**Fig. 3:**
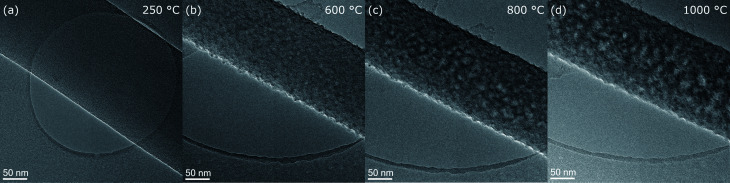
Intermediate resolution images showing the development of fiber 5 morphology at temperature steps (a) 250 °C, (b) 600 °C, (c) 800 °C and (d) 1000 °C.

The surface morphology can be studied at intermediate magnification micrographs as shown for fiber 5 in Fig. 3(a–d). In the initial state at 250 °C in Fig. 3(a), the stabilized nanofiber appears smooth with homogeneous amorphous contrast. At 600 °C, the roughness markedly increased (Fig. 3(b)) and it appears like nanosized particles are sticking out from the fiber. Also diffraction contrast arises throughout the nanofiber with brighter and darker regions with similar sizes around 5 nm. With further increase of temperature to 800 °C and 1000 °C the lateral size of the objects producing the roughness and the diffraction contrast, most probably turbostratic carbon, increases further to ≈10 nm at 800 °C and even more up to 15 nm at 1000 °C. However, the roughness itself does not continue to increase significantly between 600 and 1000 °C.

### Carbon structure analysis by HRTEM

**Fig. 4:**
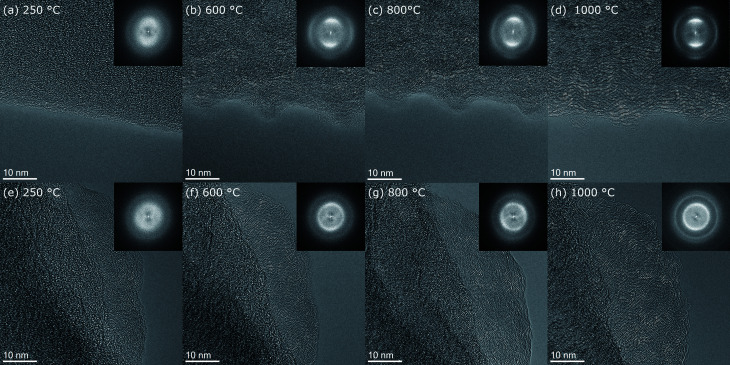
HRTEM images of the side surface of fiber 5 at temperature steps (a) 250 °C, (b) 600 °C, (c) 800 °C and (d) 1000 °C. FFT's are shown on the insets. HRTEM images and corresponding FFT's of a thin part at the tip of fiber 4 at the four temperatures (e) 250 °C, (f) 600 °C, (g) 800 °C and (h) 1000 °C.

**Fig. 5:**
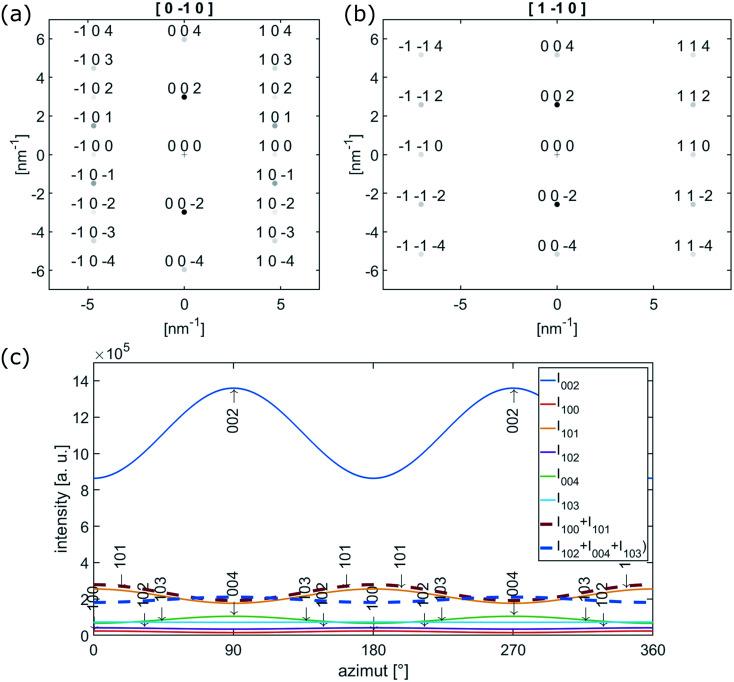
(a) [0−10] and (b) [1−10] electron diffraction pattern of single crystalline graphite. (c) Calculated azimuthal intensities for reflection up to 103 with |*g*_*hkl*_| ≤ 6.5 nm^−1^.

To evaluate the morphological changes of the nanofiber surface and the atomic structure in more detail HRTEM micrographs of the lateral surface of fiber 5 at 250 °C, 600 °C, 800 °C and 1000 °C are shown in Fig. 4(a–d). Furthermore, Fig. 4(e–h) shows HRTEM micrographs of the tip of fiber 4. For the initial state of the stabilized PAN-nanofiber at 250 °C (Fig. 4(a) and (e)) the HRTEM micrographs show an amorphous contrast and also the fast Fourier transforms (FFTs), shown in the insets, display only diffuse rings. At 600 °C (Fig. 4(b) and (f)), the ordering of the carbon atoms, mainly (002)-planes with *d*_002_ ≈ 0.35 nm, becomes visible in real space. At the lateral surface of the fiber, this ordering appears preferentially parallel to the fiber axis leading to higher intensity in the diffractogram perpendicular to the fiber axis. At 600 °C, only few planes are stacked, which corresponds to *L*_002_ in the range of few nm.^23^ Also the lateral size corresponding to *L*_10_ of these turbostratic regions is in the range of few nm.^23^ At 800 °C, lateral and stacking size is around 5 nm. The number of planes stacked and their lateral size increases markedly in the last heating step from 800 °C (Fig. 4(c) and (g)) to 1000 °C (Fig. 4(d) and (h)). Here, turbostratic regions become as large as 10 nm, in agreement with the diffraction contrast observed in the intermediate resolution images in Fig. 3(c and d). This evolution can also be followed in the FFTs of the images shown in the insets. In the initial state at 250 °C (Fig. 4(a) and (e)), only two very diffuse rings are present. At 600 °C (Fig. 4(b) and (f)), these rings get more defined and especially for the 002-ring a texture appears for the HRTEM images recorded at the side of fiber 5 (Fig. 4(b)). The 002-ring shows markedly higher intensity perpendicular to the fiber surface proving the preferential alignment of (002)-planes parallel to the surface. At 600 °C (Fig. 4(b) and (f)) and 800 °C (Fig. 4(c) and (g)), the former second diffuse ring splits into two rings. At 1000 °C (Fig. 4(d) and (h)), these two rings are clearly distinguishable. In the FFTs of the images recorded at the lateral surface of the nanofiber, the inner of these two rings with smaller scattering vectors shows high intensity parallel to the fiber axis and the outer ring has the maximum intensity perpendicular to the fiber axis like the 002-ring. Comparing the scattering vectors and relative intensities for reflections of graphite listed in Table S1,† the first subring of the second ring can be attributed to {100}- and {101}-planes, which are perpendicular to {002}-planes or form an angle of ≈72° with them (see also Fig. 5(a)).

**Fig. 6:**
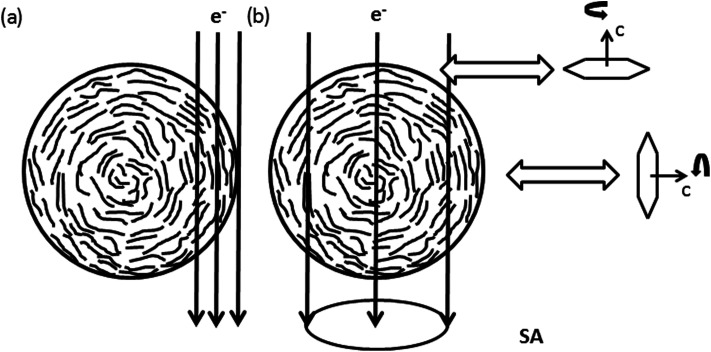
Schematic sketch of the nanofiber texture showing the preferential alignment of {002}-planes parallel to the nanofibers surface. (a) Shows conditions of HRTEM images recorded at the side of the nanofiber in Fig. 4(a)–(d). In (b) the condition for Electron Diffraction (ED) with a selected area Aperture (SA) positioned in the central part of the nanofiber, as applied for electron diffraction in Fig. 7, is shown. The rotation around the *c*-axis remains a degree of freedom within the graphitic planes.

The {102}-, {004}- and {103}-planes contribute to the second subring with *g*_*hkl*_ in the range from 5.5 to 6.5 nm^−1^. Due to their multiplicity and orientation the {102}- and {103}-planes form a rather homogenously distributed intensity in the azimuthal range. The texture observed within the second subring arises mainly from 004, the second order reflection of the (002)-planes. In the proposed structure sketched in Fig. 6(a), close to the nanofiber surface most of the graphitic (002)-planes are parallel to the electron beam and in diffraction condition. However, the rotation around the *c*-axis remains a degree of freedom and therefore the relative intensity of 002 and 004 is expected to be higher, compared to *h*0*l*- and *hkl*-reflections, which are excited for graphitic regions in 〈100〉 and 〈1−10〉 zone axis orientations only, as depicted in Fig. 5(a) and (b).

The observed variation of scattered intensities with the azimuth could be reproduced by calculations using a simple model. Starting with the [0−10] zone axis diffraction pattern as depicted in Fig. 5(a) the variety of c-axis orientation within the plane perpendicular to the electron beam is introduced by a Gaussian distribution with *σ* = 60° around the azimuthal positions of the reflections. Furthermore the relative intensities of *h*0*l*- and *hkl*-reflections are artificially decreased by a factor of 0.5 to represent relative intensities of the higher order reflections in respect to 002. The resulting intensity for reflections up to 103 (*g*_*hkl*_ ≤ 6.5 nm^−1^) is plotted as a function of the azimuthal angle in Fig. 5(c). An azimuthal angle of 0° and 180° corresponds to scattering parallel to the fiber axis, 90°and 270° perpendicular to the fiber axis. The thick dashed lines represent *I*_100_ + *I*_101_ (red) and *I*_102_ + *I*_004_ + *I*_103_ (blue), which in first approximation can be attributed to the inner and outer subring of the former diffuse second ring in the FFTs in Fig. 4(b–d). Furthermore, the graph represents the higher intensity parallel to the fiber axis for the inner subring (red) and the higher intensity of the second subring (blue) perpendicular to the fiber axis.

Enhanced ordering in the graphitic structure under the influence of high temperatures is also visible at fracture surfaces at the tip of the nanofiber pieces (Fig. 4(e–h)). In contrast to the turbostratic structures formed on the lateral surfaces no preferential orientation of the turbostratic areas can be recognized in the real space image. Confirming this observation, the FFTs shown in the insets of Fig. 4(e–h) show rings with homogeneously distributed intensity in the azimuthal range as expected for randomly oriented crystallites. With increasing temperature these rings just get sharper and more defined. The interpretation of these observations is sketched in Fig. 6. As in the central part of the nanofiber the distance to the surface is comparable in all directions, so the ordered regions in this part of the nanofiber have arbitrary orientations among each other. Thus we conclude that ordering of carbon atoms in a turbostratic form applies also to the inner parts of the nanofiber. The 002-texture on the other hand is predominantly formed close to the nanofiber surface.

### Carbon structure from electron diffraction

**Fig. 7:**
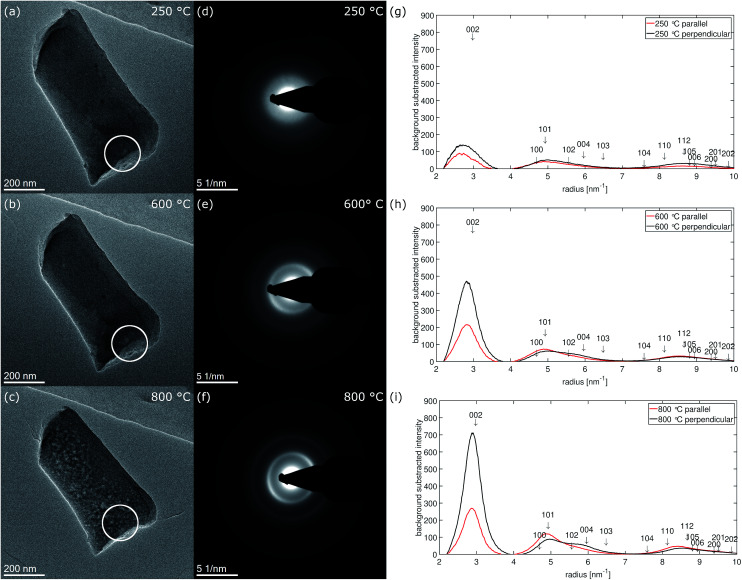
Fiber 2 selected for electron diffraction experiment at temperature stages (a) 250 °C, (b) 600 °C and (c) 800 °C. The position of the selected area aperture (SA) is marked by a white circle. (d–f) Show the corresponding electron diffraction patterns, (g–i) the background subtracted radial intensity parallel and perpendicular to the fiber axis at the corresponding temperatures.

Fig. 7 shows micrographs of fiber 2 selected for electron diffraction in (a–c) and the corresponding diffraction patterns recorded at 250 °C, 600 °C and 800 °C in (d–f). The position of the selected area aperture (SA) with a size of ≈170 nm in the intermediate image plane is displayed by a white circle. In contrast to HRTEM, in this experiment the complete thickness of the nanofiber inside the aperture contributes to the electron diffraction pattern as sketched in Fig. 6(b). As the aperture size does not include the complete nanofiber diameter, the diffraction patterns reveal a bit more the structure of the central part plus the upper and the lower surface of the nanofiber. This is complementary to the FFTs of the high-resolution images obtained at the fiber surface on the side of the nanofiber, which is excluded by the aperture. The areas selected for recording the pattern were chosen close to the broken tip of the nanofiber to contain only small fraction of fracture edge, but also allowing a precise location of the SA at different stages of the *in situ* experiment in the TEM.

At 250 °C, three diffuse rings can be recognized in the diffraction pattern (Fig. 7(d)). At 600 °C (Fig. 7(e)), mainly the first, the 002-ring, becomes more defined and already starts to exhibit higher intensity for scattering vectors perpendicular to the fiber axis. For the second ring the intensity increase at low scattering vectors ≈ 4.6 nm^−1^ becomes sharper while the decay to larger scattering vectors stays rather diffuse. At 800 °C (Fig. 7(f)), the texture in the 002-ring becomes more pronounced and the former second diffuse ring splits into two distinguishable rings, of which the first one at lower scattering vectors is of higher intensity.The evolution of the diffraction patterns as described above can be followed in the background subtracted radial intensity, which was extracted from the diffraction patterns plotted in Fig. 7(g–i). An exponential background in the form of eqn (1)

1 *a*_1_ e^−*b*_1_(*μ*_1_−*x*)^ + *a*_2_ e^−*b*_2_(*μ*_2_−*x*)^ + *d*

was fitted to the decaying intensity of the primary beam. To accommodate the texture effects we extracted the radial intensity over an azimuthal range of ±20° parallel and perpendicular to the fiber axis. This corresponds to an azimuthal range from 215° to 255° (perpendicular) and from 305° to 345° (parallel) in the diffraction patterns in Fig. 7(d–f). 0° and 360° represent the 12 o'clock position and the azimuth increases clockwise. The radial intensities are plotted in Fig. 7(g–i). In all plots arrows indicate the positions of reflections of crystalline graphite and their vertical positions represent the relative intensities of these reflections as listed in Table S1.†

Within the diffraction patterns, only the first ring originates from a single reflection, which is 002. The position of its maximum at 800 °C is found at ≈2.84 nm^−1^ corresponding to a *d*_002_-spacing of 352 pm. The second ring already contains five reflections, 100, 101, 102, 004 and 103. The sharp increase of intensity at low scattering vectors can be attributed to the rather closely packed 100 (4.694 nm^−1^*I*_rel_ ≈ 3%) and 101 (4.925 nm^−1^*I*_rel_ ≈ 18%). Among the five reflections contributing the second ring 101 is by far the most intense reflection. The diffuse decay to larger scattering vectors can be attributed to more broadly distributed 102 (5.560 nm^−1^*I*_rel_ = 3%), 004 (5.961 nm^−1^*I*_rel_ = 7%) and 103 (6.482 nm^−1^*I*_rel_ = 5%) reflections. At 800 °C, two separate rings become distinguishable in the formerly second ring, which can be attributed to these previously described two groups of reflections. We attribute the first subring to 001 and 101, the second subring to 102, 004 and 103 reflections. As the first subring is dominated by 101, it shows higher intensity parallel to the fiber axis. For the second subring the highest intensity is observed for scattering perpendicular to the fiber axis, which can only arise from 004. Both observations can be explained by the calculated azimuthal intensity distribution shown in Fig. 6(c). The same texture also influences the relative intensity of the reflections under the third main ring in the range from ≈7.5 nm^−1^ to ≈10 nm^−1^. In this range 110 (5.4%) and 112 (8.7%) are the most intense reflections and show higher intensity parallel to the fiber axis.

The texture evolution can be followed by comparing the background subtracted radial intensities parallel and perpendicular to the fiber axis. At 250 °C, no significant difference between scattering parallel and perpendicular (b) to the fiber axis is noticed. At 600 °C, the 002-intensity is already much stronger perpendicular to the fiber and lower parallel to the fiber axis. Perpendicular to the fiber axis for the second ring a hump between 5.5 and 6 nm^−1^ can be recognized next to the reduced main peak, which is formed by the 101 reflection (4.925 nm^−1^*I*_rel_ ≈ 18%) with little contributions of 100 (4.694 nm^−1^*I*_rel_ ≈ 3%). This hump can be explained with the 004 peak (5.952 nm^−1^), which is the second order reflection of 002. Parallel to the fiber axis 004 is less intense and the main peak is dominated by 101 and shows only long decay towards larger scattering vectors. At 800 °C, this behavior becomes even more pronounced. Nevertheless, the texture effect in the two subrings of the second ring in the diffraction patterns in Fig. 7(e) and (f) is much weaker than in the FFTs of the HRTEM micrographs recorded at the side surface of fiber 5 in Fig. 4(b–d).

**Fig. 8:**
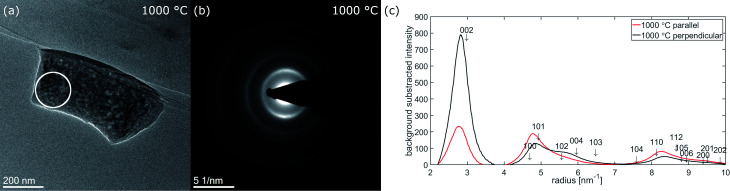
(a) Fiber 2 in slightly different orientation at 1000 °C with the position of the SA for diffraction marked by a the white circle (b) electron diffraction pattern at 1000 °C (c) extracted background substracted radial intensities parallel (red) and perpendicular (black) to the fiber axis subtracted radial intensity parallel and perpendicular to the fiber axis.

During the last heating step up to 1000 °C, the nanofiber selected for diffraction moved on the heating chip, so the orientation of the fiber and the position of the selected area aperture are not identical to the previous measurements at the other temperatures. Nevertheless, a diffraction pattern was recorded and is displayed in Fig. 8 together with the corresponding micrograph as well as with the radial intensity extracted parallel and perpendicular to the fiber axis. According to the different fiber orientation, the angular ranges for extracting the radial intensities parallel and perpendicular to the fiber axis changed to 175–215° (parallel) and 265–305° (perpendicular). The texture effects at 1000 °C are more pronounced compared to the results at lower temperatures, with almost equal intensity of 002 and 100 + 101 parallel to the fiber axis. The presence of texture in diffraction arising from a volume close to the center of the fiber means the texture is also present inside the fiber. The observation of diffraction rings up to 10 nm^−1^ correlates with an extended graphitic order. This is supported by Raman spectra measured *ex situ* on fiber mats carbonized for 10 h in argon atmosphere at the same temperatures shown in Fig. S3.† The spectra are scaled to the D-band maximum at around 1350 cm^−1^. An increase of G-band intensity around 1580 cm^−1^ with increasing carbonization temperature is clearly visible.

## Discussion

*In situ* TEM carbonization experiments provide an approach to investigate the morphological and structural changes during carbonization based on observations of individual nanofibers. In combination with spectroscopic methods such as EELS and energy dispersive X-ray spectroscopy (EDX) available in the TEM also the evolution of the chemical composition can be investigated *in situ*. By tracking the evolution of fiber diameter and structure of the same nanofiber up to temperatures of 1000 °C, statistical scattering, which stems from comparing different fibers in *ex situ* experiments, can be avoided. In fact, analysis of SEM images (Fig. S1 and S2†) resulted in a broad distribution of fiber diameters. *In situ* TEM enables to measure the shrinkage in diameter on the identical fiber at any time during an experiment. After 1000 °C, we observed shrinkage in the range of 15 to 20% in diameter, which correlates to a loss in cross sectional area in the range from 27 to 35%. Musiol *et al.* reported a weight loss of ≈39% for carbon microfibers and 55% for carbon nanofibers measured by thermogravimetry.^18^ Translating the shrinkage observed in our study into a mass loss, the shrinkage in our experiment is comparable. Differences could arise from different fibers used at the initial state and the formation of porosity, which could increase the mass loss with respect to shrinkage of fiber diameter.

By intermediate TEM magnification micrographs, we observed a marked increase of the nanofiber roughness, especially in the first heating step to 600 °C. The surface roughening was accompanied by diffraction contrast arising inside the fibers. Roughening of carbon nanofibers derived from electrospun PAN upon graphitization at higher temperatures of 1500 and 2800 °C was also reported by Kurban *et al.*^27^ Bennett used diffraction contrast in dark-field TEM to image regions of turbostratic graphite in carbon microfibers treated at temperatures.^21^ In our study, we observe regions with sizes around 5 nm after 600 °C and up to 10 nm after 800 °C or even up to15 nm at 1000 °C. These sizes agree with our HRTEM micrographs, where we estimate the size of ordered regions within the graphitic plane (*L*_10_ or *L*_a_) and perpendicular to them (*L*_002_ or *L*_c_) of few nm at 600 °C, up to 5 nm at 800 °C and up to 10 nm at 1000 °C. So the interpretation as diffraction contrast arising from turbostratic carbon is valid. A slightly higher measure in diffraction contrast is not surprising as the field of view and the statistics are better the image resolution poorer. In our case the estimated sizes in and out of plane are comparable. Laffont *et al.* retrieved *L*_10_ ≈ 3–4 nm by Warren Bodenstein formula and *L*_002_ ≈ 1 nm retrieved from the Scherrer equation, both from XRD results.^23,33^ Kim *et al.* achieves similar results for *L*_c_ from XRD *via* the Scherrer equation.^34^ In their work the values for *L*_10_ (or *L*_a_) extracted from Raman spectra *via* the Knight and White equation are also larger than the values of *L*_002_.^34,35^ Deviations of sizes measured with different methods can be expected. In addition results are sensitive to experimental conditions and the initial fibers used.

In agreement with literature we observed a preferential alignment of the graphitic planes with the fiber axis.^18,21,22,27,34^ This texture is confirmed by the FFTs of the HRTEM images as well as the electron diffraction patterns. Comparing our HRTEM micrographs to others the graphitic regions in our experiment appear more disordered with planes more bend and also the borders of the ordered regions are less defined.^18,21,22,27,34^ Most similarity is found with the micrographs published by Kim *et al.* However clearly in our micrographs a higher number of planes stacked corresponding to higher *L*_c_ is visible.^20^ The curvature of graphitic planes observed in HRTEM is well reflected by the rather broad peaks in the azimuthal distribution within the diffraction rings in the FFTs and the electron diffraction patterns. We achieved good agreement of the experimentally observed azimuthal intensity distribution with calculated intensities based on a simple model, which correlates to a variation in orientation of the graphitic (002)-planes. A different degree of texture for HRTEM-micrographs recorded at the fiber surface, the broken tip of the nanofiber and electron diffraction was observed. This can be explained with the volume probed in each experiment. In the HRTEM micrographs of the fiber surface in Fig. 4(a–d) only the structure of the surface up to a depth of ≈30 nm is probed. With the texture proposed in Fig. 5(a), similar to that suggested by Bennet for microfibers, most of the (002)-planes are parallel to the electron beam in this region of the fiber resulting in enhancement of 002-reflection compared to the averaged structure.^11^ In the electron diffraction experiment on the other hand, the selected area aperture is positioned more central to the fiber axis (Fig. 5(b) and 7(a–c)) so diffraction arises from the central volume of the fiber plus the upper and lower surface. In this part of the fiber (002)-planes are expected in perpendicular orientation to the electron beam and therefore not in diffraction condition. The aperture with a diameter of ≈170 μm in the intermediate image plane cuts of regions close to the surface which contribute to the HRTEM-micrographs taken at the fiber surface. Nevertheless texture effects are observed, underlining that, even with some distance to the nanofiber surface, the texture still is present. However, the HRTEM-micrographs recorded at the broken tip of a nanofiber don't show texture. Within the region chosen there, the turbostratic carbon is randomly oriented. We propose to apply both HRTEM and diffraction as these two techniques are complementary in respect to the volume probed. While for the analysis of nanofibers designed for mechanical applications the knowledge about the structures in the fiber cores is crucial, properties of the surfaces and surface near regions are the most relevant for the application of nanofibers in electrodes for metal–air batteries, fuel cells or electrocatalysis.

Quantitative analysis of the diffraction data showed that the 002 peak stabilizes around 2.84 nm^−1^ at 800 °C and 1000 °C corresponding to a *d*_002_-spacing of 352 pm compared to 335 pm for graphite.^25^ Laffont *et al.* and Kim *et al.* reported during stabilization like our fiber mat, after 1000 °C heat treatment part of the nitrogen remains in the fiber.^20,26^ However, in the case of an *in situ* TEM experiment an influence of UHV compared to inert gas atmosphere in an *ex situ* experiment on *d*_002_-values of 349 pm and 357 pm respectively for PAN derived carbon fibers.^23,34^ Our result is comparable to both of them. Kim *et al.* attribute the stacking distance to the presence of quaternary nitrogen.^20^ Laffont *et al.* showed that in fiber set 2, which was allowed to shrink the nitrogen content is possible. If the microscope is equipped with a spectrometer, the chemical composition can be tracked by EELS or EDX during the *in situ* experiment.

As shown above the techniques available in a TEM allow a broad spectrum of analysis to study the carbonization of PAN-derived nanofiber during an *in situ* experiment. Results are at least comparable to *ex situ* experiments. However, it has to be kept in mind that a complete reproduction of *ex situ* experiments inside the TEM is not possible as parameters such as heating rate and atmosphere affect the final properties of the nanofibers.^34,36^ Combined *in situ* heating experiments under gas flow, which are possible nowadays, could help to circumvent this issue. During the first 250 °C heating step some influence of the electron beam was evidenced from the substantial difference in fiber diameter shrinkage, depending on the exposure of the nanofiber to the electron beam. The latter might be attributed to the interaction of the electron beam with residual gases in the microscope and the applied heating of the sample.^37–39^ The fibers with higher exposure to the electron beam under UHV conditions at 250 °C show faster shrinkage. Also an influence on the surface roughness is possible. This could be controlled by a comparison with additional *ex situ* experiments on identical locations. In any case, the beam exposure should be kept to a minimum, which we did for the rest of the fibers. With respect to the main subject of research – the formation of graphitic structures in the fibers depending on carbonization temperature – this confinement does not preclude the *in situ* method for these objectives.

With the combination of TEM at low, intermediate and high magnification and electron diffraction we were able to follow the shrinkage of fiber diameter and the development of the surface morphology as well as the carbon structure during the *in situ* experiment. In combination with complementary *ex situ* experiments, the *in situ* experiments can contribute to an overall picture of the carbonization process for polymer-derived carbon nanofibers. Thus, the *in situ* carbonization TEM technique provides a promising approach which is currently just at the beginning of its development.

## Conclusions

Dimensional changes, surface morphology as well as the structure of PAN-derived carbon nanofibers were investigated by *in situ* TEM during their carbonization up to 1000 °C. Shrinkage in diameter as observed on individual nanofibers over the whole temperature range mounts up to 20% reduction of the initial size. No marked influence of the initial nanofiber diameter on its relative reduction was detected. Enhanced shrinkage – in particular at the 250 °C temperature stage – was indicated for nanofibers, which were subject to intensive exposure to the electron beam. As a second major result, intermediate resolution TEM imaging clearly revealed the roughening of the surface, which is beneficial for catalytic applications. Moreover, intermediate TEM also revealed the transition from amorphous contrast to an increased diffraction contrast on the length scale of about 5 nm at 600 °C to ≈10 nm at 800 °C and even up to 15 nm at 1000 °C. HRTEM showed a similar behavior with slightly smaller sizes. Furthermore, HRTEM and its FFT showed the presence of turbostratic ordered regions for temperatures 600 °C and above, with preferential alignment of the (002)-planes parallel to the fiber axis on the lateral surfaces. The more graphitic structure should increase the electronic conductivity of the nanofibers for electrochemical applications. Finally, electron diffraction showed that the same texture continues inside the nanofiber. The observation of higher order reflections confirms the growth of the ordered regions. Based on quantitative evaluation of the 002-ring in the electron diffraction patterns a *d*_002_ spacing of ≈352 pm was determined at the end of the experiment. Overall, *in situ* TEM, with its possibility of different imaging techniques, diffraction and also spectroscopic methods provides a powerful tool to study the carbonization of PAN-derived nanofibers on identical locations at any time during the experiment.

## Conflicts of interest

There are no conflicts to declare.

## Supplementary Material

RA-009-C8RA10491C-s001
