# Supplementary material for: The carbonization of polyacrylonitrile-derived electrospun carbon nanofibers studied by in situ transmission electron microscopy
Source: RSC Adv. 2019 Feb 21;9(11):6267–77. doi: 10.1039/c8ra10491c (PMC9060947; doi:10.1039/c8ra10491c)
Supplement: RA-009-C8RA10491C-s001 [file RA-009-C8RA10491C-s001.pdf]

Electronic Supplementary Material (ESI) for RSC Advances.
This journal is © The Royal Society of Chemistry 2019
Please do not adjust margins

Journal Name

ARTICLE

The carbonization of Polyacrylonitrile-derived electrospun carbon nanofibers studied by *in situ* Transmission Electron Microscopy

Received 00th January 20xx,
Accepted 00th January 20xx

DOI: 10.1039/x0xx00000x

www.rsc.org/

Supplementary Information

Roland Schierholz[a] *, Daniel Kröger[a], Henning Weinrich[a,b], Hermann Tempel[a], Hans Kungl[a], Joachim Mayer[c,d] and Rüdiger-A. Eichel[a,b]

[a] *Institute of Energy and Climate Research, Fundamental Electrochemistry (IEK-9), Forschungszentrum Jülich GmbH, 52425 Jülich, Germany*
[b] *Institute of Physical Chemistry, RWTH Aachen University, 52074 Aachen, Germany*
[c] *Ernst Ruska-Centre for Microscopy and Spectroscopy with Electrons, Forschungszentrum Jülich GmbH, 52425 Jülich, Germany*
[d] *Central Facility for Electron Microscopy, RWTH Aachen University, 52074 Aachen, Germany*

† Footnotes relating to the title and/or authors should appear here.
Electronic Supplementary Information (ESI) available: [details of any supplementary information available should be included here]. See DOI: 10.1039/x0xx00000x

Please do not adjust margins

Table S1: Spacing of lattice planes d_{hkl} in real space, length of corresponding diffraction vector g_{hkl} in reciprocal space and the relative intensity for graphite powder calculated from ICSD-file 76767 up to hkl = 006. Also given is the azimuthal position of the diffraction vector assuming a single crystal of graphite with (002) planes parallel to the fiber axis and the electron beam. The last two columns represent, if the corresponding intensity is expected to increase ↑ , stay constant - or decrease ↓ if extracted from an azimuthal range ± 20 ° perpendicular ⊦ or parallel | | to the fiber axis.

h	k	l	d_{hkl} [pm]	$\|g_{hkl}\|$ [1/nm]	I_{rel} [%]	m	azimut [°]			$I_{⊦}$	$I_{\|\|}$
Zone [0-10]											
0	0	2	336	2.985	100	2	90		270	↑	↓
1	0	0	213	4.695	3.5	6	0		180	↓	↑
1	0	1	203	4.926	19.4	12	342	18	162	↓	↑
1	0	2	180	5.587	3.4	12	328	32	148	↓	↑
0	0	4	168	5.952	7.7	2	90		270	↑	↓
1	0	3	154	6.494	6.5	12	316	44	136	-	-
1	0	4	132	7.576	0.9	12	308	52	128	-	-
1	0	5	113.6	8.807	2.0	12	302	58	122	-	-
0	0	6	111.9	8.942	1.6	2	90		270	↑	↓
Zone [1-10]											
0	0	2	336	2.985	100	2	90		270	↑	↓
0	0	4	168	5.952	7.7	2	90		270	↑	↓
1	1	0	123	8.130	6.7	6	0		180	↓	↑
1	1	2	115.7	8.659	10.6	12	328	32	148	↓	↑
0	0	6	111.9	8.942	1.6	2	90		270	↑	↓

This journal is © The Royal Society of Chemistry 20xx

Please do not adjust margins

Please do not adjust margins

Please do not adjust margins

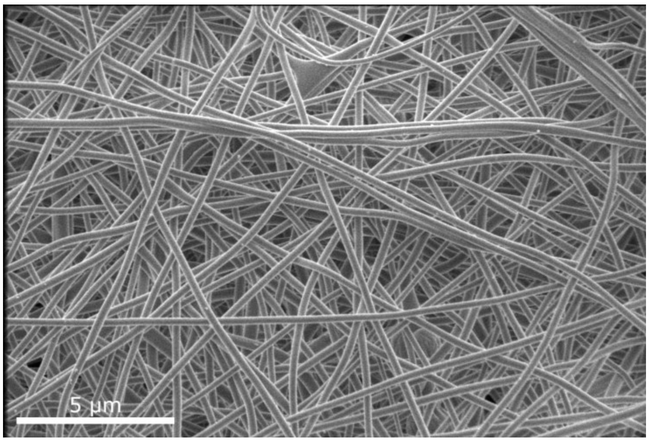

Figure S1: SEM image of a fiber mat oxidatively stabilized at 250 °C, which was the starting material for the *in situ* experiment.

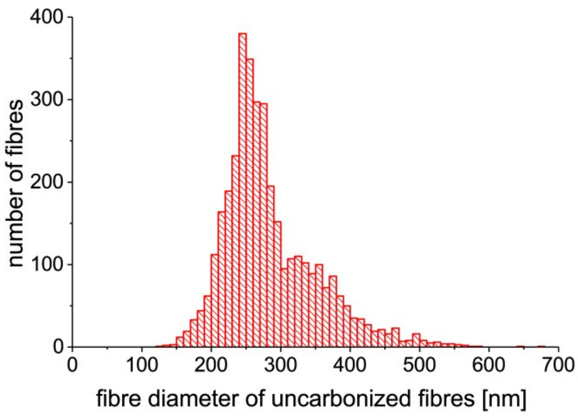

Figure S2: Distribution of fiber diameters of oxidatively stabilized fiber mats measured from SEM micrographs.

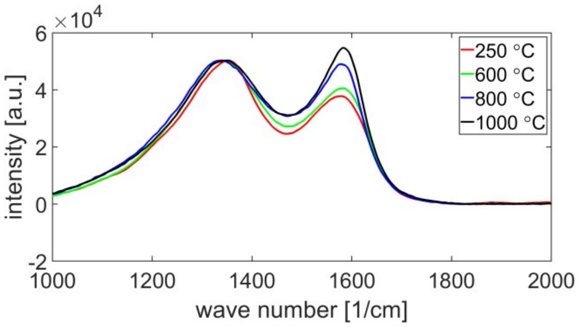

Figure S3: Raman spectra measured *ex situ* on fiber mats after oxidative stabilization at 250 °C and carbonized in a tube furnace under Argon flow for 10 h at 600 °C, 800 °C and 1000 °C.

Please do not adjust margins

Please do not adjust margins

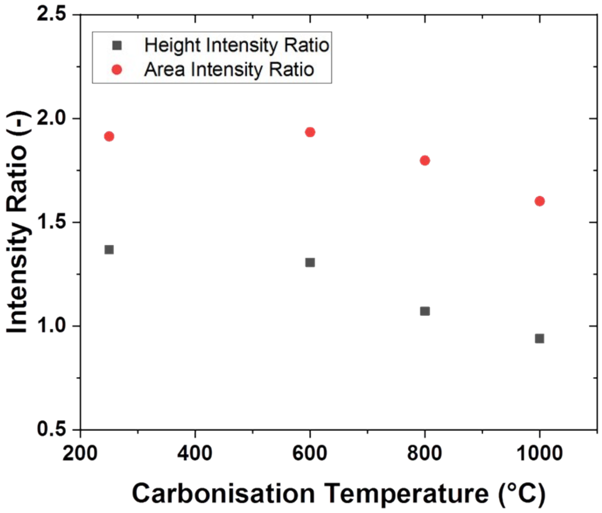

Figure S4: Intensity ratios of the D-band (≈ 1350 cm^{-1}) over the G-band (≈ 1550 cm^{-1}) calculated using the peak area (red circles) and just the maximum intensity (balck squares).

This journal is © The Royal Society of Chemistry 20xx

Please do not adjust margins
